# Unraveling land-use carbon-pollution co-evolution: A dynamic coordination framework with causal pathways and spatial zoning

**DOI:** 10.1016/j.isci.2026.116520

**Published:** 2026-06-27

**Authors:** Xue Zhao, Bilin Shao, Jia Su, Ning Tian, Wei Zhao, Xinyu Liu

**Affiliations:** 1 School of Management, Xi’an University of Architecture and Technology, Xi’an 710055, China

**Keywords:** synergistic evolution index, land-use carbon emissions, Bayesian network, DBSCAN clustering, spatial environmental governance

## Abstract

The co-evolution of land-use carbon emissions and fine particulate matter (PM_2.5_) pollution is increasingly used to inform differentiated regional governance, yet its temporal asynchrony, spatial clustering, and causal pathways remain insufficiently quantified. This study develops a multi-stage framework to diagnose carbon-pollution synergy across 113 district-county units in the Fenwei Plain, China, from 2013 to 2023. A process-sensitive synergistic evolution index (SEI) is constructed by integrating dynamic time warping (DTW) and a coupling coordination degree (CCD) metric to capture lag-aware co-movement and coordination. The results reveal pronounced spatial polarization in synergy performance, with significant global and local clustering patterns. Bayesian network analysis further identifies heterogeneous pathways and highlights the mediating role of urban expansion in linking ecological and anthropogenic drivers to synergy outcomes. Density-based clustering delineates four governance zones with distinct stability and enhancement potential, providing an empirical basis for spatially tailored carbon-pollution mitigation strategies.

## Highlights

• An SEI integrating DTW and CCD captures lag-aware carbon-PM2.5 co-evolution
• Carbon-pollution synergy improved but remained spatially uneven
• Bayesian networks reveal urban-expansion-mediated synergy pathways
• DBSCAN delineates four spatially differentiated governance regimes

## Graphical abstract

**Figure:**
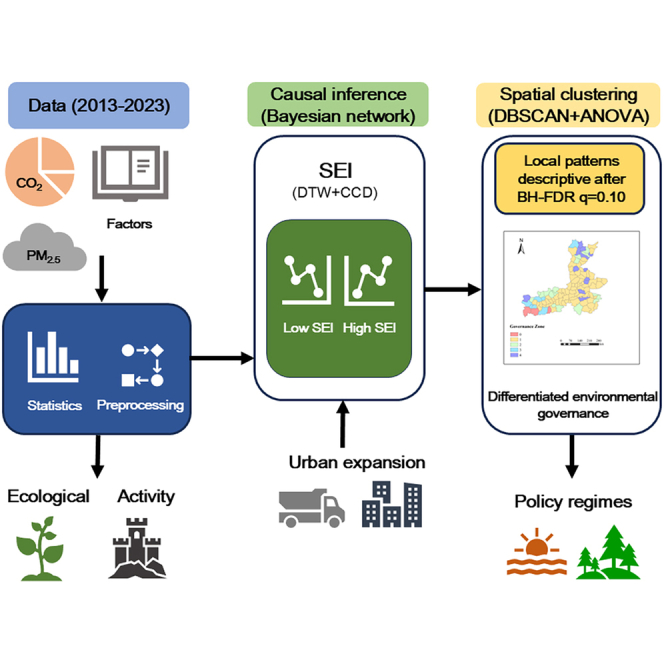

## Teaser

Atmospheric science; Environmental science; Global change

## Introduction

Coordinated control of carbon dioxide (CO_2_) and air pollutants has become a mainstream policy paradigm because combustion activities co-emit climate and air quality forcers. Comparative assessments indicate that climate mitigation can deliver substantial air quality and health co-benefits that offset a meaningful share of compliance costs.^1^ Global multi-model evidence further confirms that greenhouse gas mitigation improves future fine particulate matter (PM_2.5_) exposure and reduces premature mortality, with monetized health gains comparable to mitigation costs under Paris-consistent pathways and sizable co-benefits across regions and sectors.^2^^,^^3^^,^^4^^,^^5^ In China, integrated packages linking carbon peaking with clean-air controls have driven sharp declines in major pollutants since 2013 while reshaping the energy mix.^6^ Sectoral and scenario analyses also suggest that coal retirement and earlier CO_2_ peaking can yield additional health and air quality benefits, and that net climate-air outcomes remain favorable even when end-of-pipe controls marginally raise CO_2._^7^^,^^8^^,^^9^ However, at the urban scale, rapid industrialization and expansion continue to produce high carbon intensity and elevated pollutant concentrations, implying spatially heterogeneous prospects for carbon-pollution synergy.^10^^,^^11^ At the national scale, decomposition evidence for China suggests that economic output is the dominant driver of long-term CO_2_ growth, whereas energy intensity provides the strongest offsetting effect.^12^ At the regional scale, assessments of land-use carbon emissions (LUCE) further reveal a stable LUCE spatial correlation network and carbon-balance zoning patterns in Jiangxi Province.^13^ Yet, few studies have explicitly linked such land-based carbon diagnostics to ambient air pollution (especially PM_2.5_) to characterize their coupled evolution and spatial spillovers at policy-relevant urban scales.

Building on this policy context and the need to connect land-based carbon diagnostics with air quality outcomes, a land-use perspective is adopted, because development decisions reshape source-sink dynamics, technology choices, mobility demand, and dispersion environments.^14^ PM_2.5_ remains a dominant environmental health risk and frequently exceeds the World Health Organization (WHO) 2021 guideline of 5 μg m^−3^.^15^ Recent assessments highlight persistent exposure burdens despite improvements, while new exposure datasets harmonize satellite retrievals, chemical-transport modeling, and monitoring data to provide temporally consistent sub-national PM_2.5_ estimates with quantified uncertainty.^16^ In parallel, nighttime lights (NTLs) are validated proxies for anthropogenic activity and are widely used to spatialize fossil-fuel CO_2_ emissions and monitor urbanization.^17^^,^^18^ More broadly, recent national-scale environmental risk mapping has demonstrated that integrating multi-source remote sensing indicators with proxies of human activities and data-driven models can substantially improve the identification of pollution hotspots and their environmental constraints.^19^ In this study, NTL data, denoted as Nightlight in tables, figures, and code, are used to characterize urban activity intensity and anthropogenic disturbance. Considering land-use CO_2_ together with PM_2.5_, therefore, provides a responsive lens for examining activity-emission-exposure linkages under structural and regulatory change.^20^

Most existing synergy diagnostics, including correlations, elasticities, composite indices, and the coupling coordination degree (CCD), enable cross-regional comparisons but are often limited in capturing phase lags and coordination volatility during policy-driven transitions.^21^^,^^22^^,^^23^ Synergy is inherently dynamic and feedback-sensitive because emission changes can precede ambient PM_2.5_ improvements due to secondary formation and meteorological modulation, producing temporal misalignment that static constructs cannot represent well.^24^^,^^25^ To address these limitations, a synergistic evolution index (SEI) is developed by integrating dynamic time warping (DTW) with CCD. DTW provides process-aware temporal alignment to detect lagged co-evolution, while CCD preserves an interpretable measure of system coordination across subsystems.^26^^,^^27^^,^^28^ Because urban expansion can modify synergy through emissions growth, activity reallocation, and dispersion conditions, and cross-sensor NTL harmonizations improve trend fidelity for urban diagnostics and CO_2_ spatialization, the joint carbon-pollution process is expected to exhibit nonlinear thresholds, scale effects, and spatial spillovers.^29^^,^^30^

Beyond measurement, explaining why synergy emerges or fails requires a causal lens and an explicit treatment of spatial heterogeneity. Bayesian networks (BNs) offer a probabilistic graphical framework that captures conditional dependencies and supports interventional comparisons under uncertainty, while retaining interpretability and the capacity to encode expert knowledge.^31^^,^^32^ Recent applications have demonstrated their utility for environmental mechanism diagnosis and decision support in complex air quality contexts.^33^^,^^34^^,^^35^ For governance design, spatial heterogeneity must also be translated into operational regimes. Density-based spatial clustering of applications with noise (DBSCAN) can identify irregular and non-contiguous synergy regimes from SEI profiles and detect local anomalies that merit targeted intervention.^36^^,^^37^ DBSCAN has been applied to isolate pollution-related clusters in air quality contexts, and spatial statistics such as local indicators of spatial association (LISA) and hotspot statistics provide complementary tools for diagnosing spatial structure.^38^^,^^39^^,^^40^ Recent advances in high-resolution spatial mapping further highlight that fine-scale pollution gradients can shape exposure assessment and causal inference.^41^

**Figure 1:**
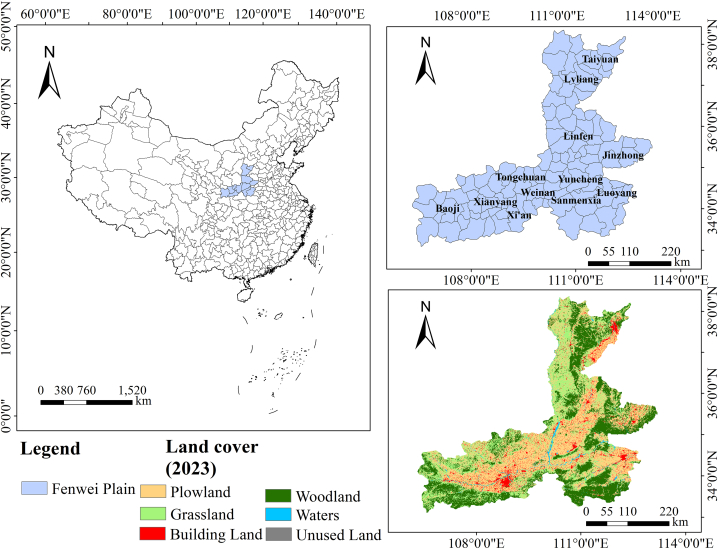
Geographic location of the Fenwei Plain Scale bars indicate distance in kilometers.

The Fenwei Plain was selected as a representative case region to evaluate carbon-pollution co-evolution and spatially differentiated governance. As shown in Figure 1, the region spans central and southern Shanxi, the Guanzhong region of Shaanxi, and parts of western Henan, comprising 113 district-county administrative units while retaining the prefecture-level structure for aggregation.^42^ It is a major energy-rich and industrial hub, having undergone intensive urban expansion, accelerated building-land growth, and continued encroachment on agricultural and ecological land, resulting in an increasingly human-modified and heterogeneous land system.^43^ Annual mean PM_2.5_ concentrations remain among the highest in China, reflecting the combined influence of energy-intensive industries, coal-dominated energy use, topographic enclosure, and unfavorable meteorology, highlighting the policy relevance for evaluating synergistic mitigation pathways under China’s dual-carbon and clean-air governance goals.^44^ Land-use-related carbon emissions were calculated using established land-use emission and absorption coefficients derived from the Intergovernmental Panel on Climate Change (IPCC) land-use carbon accounting guidance and related literature.^45^ The coefficients are listed in Table 1, and the full data sources, preprocessing procedures, and model implementation are detailed in the STAR Methods.

**Table 1:** Carbon emission coefficients for different land-use types

Land-use types	Carbon emission coefficients (t·hm^−2^)
Plowland	0.497
Woodland	−0.581
Grassland	−0.021
Waters	−0.253
Building land	5.390
Unused land	−0.005

Coefficients are expressed in t·hm^−2^. Positive values indicate carbon emissions, whereas negative values indicate carbon sequestration.

In response to these regional and methodological challenges, this study develops a unified multi-stage analytical framework to quantify dynamic carbon-pollution synergy, identify mechanism-relevant pathways, and support spatially differentiated governance. The main contributions are as follows.

1. A process-sensitive SEI integrating temporal alignment and coordination. SEI couples DTW with CCD to jointly capture lagged co-movement and coordinated development, providing a process-aware synergy metric that reveals nonlinear trajectories and coordination instability between land-use carbon emissions and PM_2.5_.
2. A mechanism-oriented causal analysis module based on BNs. A domain-informed BN structure is constructed by incorporating key drivers and compound variables, including urban expansion rate and forest land ratio, to characterize multivariate nonlinear linkages. Interventional inference and scenario contrasts are further used to quantify how joint carbon and pollution pressures are associated with shifts in SEI levels under uncertainty.
3. A data-driven spatial zoning strategy for synergy-based governance regimes. DBSCAN is applied to SEI profiles to delineate irregular and non-contiguous response regimes that reflect intrinsic synergistic behaviors beyond administrative partitions, thereby providing an empirical basis for differentiated mitigation strategies across heterogeneous subregions.

## Results

### Spatiotemporal evolution of land-use carbon emissions and air pollution

**Figure 2:**
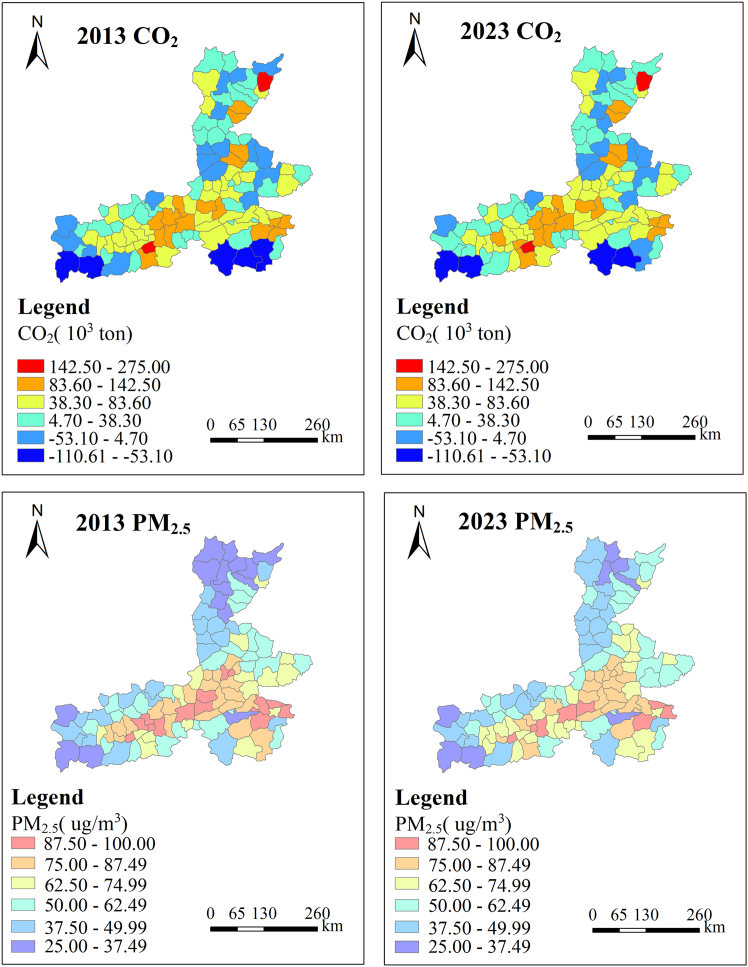
Spatiotemporal evolution of land-use carbon emissions and air pollution in the Fenwei Plain for 2013 and 2023 Scale bars indicate distance in kilometers.

As shown in Figure 2, the Fenwei Plain exhibited a pronounced decline in PM_2.5_ concentrations from 2013 to 2023, while land-use-related CO_2_ emissions displayed a more heterogeneous and locally divergent pattern. The PM_2.5_ maps indicate that high-concentration areas along the Fenhe Valley and the Weihe Basin weakened overall, consistent with region-wide air quality improvement. However, the CO_2_ panels do not support a uniform regional decrease: several subregions show stable or increasing emissions, with visible local increases in parts of Baoji and Luoyang, indicating that decarbonization progress has been uneven across the study area. The intermediate-year maps for 2015, 2018, and 2020 (Figure S1) further confirm that the spatiotemporal trajectories are non-monotonic in some locations, where emission rebounds or persistence co-occur with continued PM_2.5_ reductions.

Spatially, the northern hilly belt and the southern mountainous ecological zones remain characterized by relatively low emission and low pollution levels, whereas urban-industrial corridors retain higher intensities and stronger variability. Overall, the results suggest that regional air quality gains were broadly achieved, but carbon-pollution co-evolution is characterized by spatially differentiated pathways, leading to an increasingly visible mismatch between emission intensity and pollution pressure in specific subregions. This heterogeneity motivates differentiated, adaptive governance strategies rather than a uniform “co-decline” interpretation.

### Synergy measurement

#### DTW-based temporal alignment

**Figure 3:**
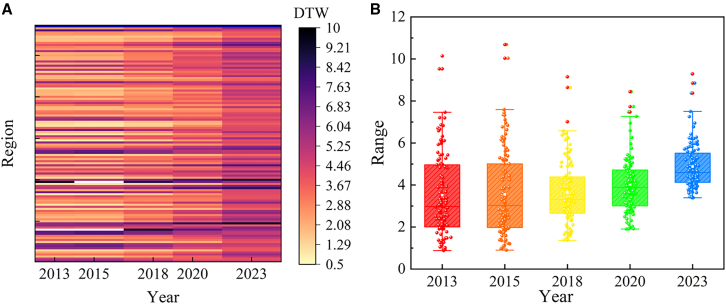
Dynamic time series analysis based on DTW (A) DTW result heatmap. (B) Yearly distribution of DTW distances. The box represents the interquartile range, the center line represents the median, the whiskers represent the non-outlier range, and each point represents one district-county unit.

Temporal dissimilarity between land-use carbon emissions and air pollution was quantified using a sliding-window DTW algorithm for 2013–2023. As shown in Figure 3A, DTW distances remained spatially heterogeneous during the study period, indicating persistent temporal misalignment between the two series in several district-county units. Figure 3B further shows that the DTW distribution did not exhibit a uniform monotonic decline, suggesting uneven temporal synchronization rather than region-wide alignment improvement. Overall, the DTW results indicate persistent phase offsets in the carbon-pollution co-evolution process, supporting the integration of DTW-based temporal alignment with CCD-based coordination in the SEI framework.

#### CCD-based coordination

**Figure 4:**
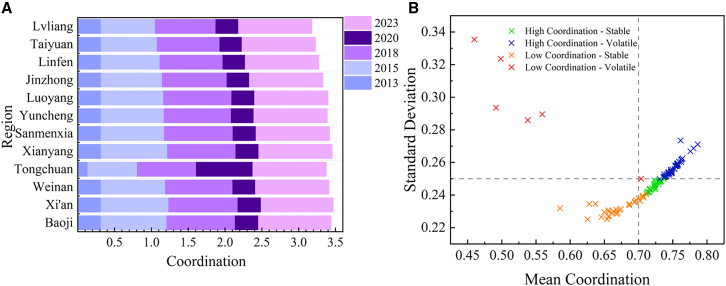
CCD analysis of carbon-pollution coordination (A) Spatial distribution of carbon-pollution coupling coordination degree. (B) Two-dimensional scatterplot of the mean and standard deviation of the coupling coordination degree.

Figure 4A shows an overall increase in city-level CCD during 2013–2023, indicating improving coordination between land-use carbon emissions and air pollution control. Xi’an, Taiyuan, and Xianyang remained highly coordinated, whereas Lvliang and Yuncheng increased more slowly. The mean-standard deviation (mean-SD) quadrant plot in Figure 4B further highlights spatial differentiation: most cities fall into the high-coordination-stable or high-coordination-fluctuating groups, while a subset remains low-coordination-fluctuating and may warrant prioritization.

Although CCD is informative for aggregate trends, it mainly reflects equilibrium coordination and is less sensitive to temporal misalignment and short-term volatility. In this study, several cities show high mean CCD but large dispersion and are classified as high-coordination-fluctuating, suggesting unstable coordination rather than persistent synergy. To address this limitation, SEI is introduced as a complementary metric. By integrating DTW-based alignment with CCD-based coordination, SEI captures both trend synchronization and system-level coupling, enabling a more dynamic assessment.

#### Construction and interpretation of SEI

**Figure 5:**
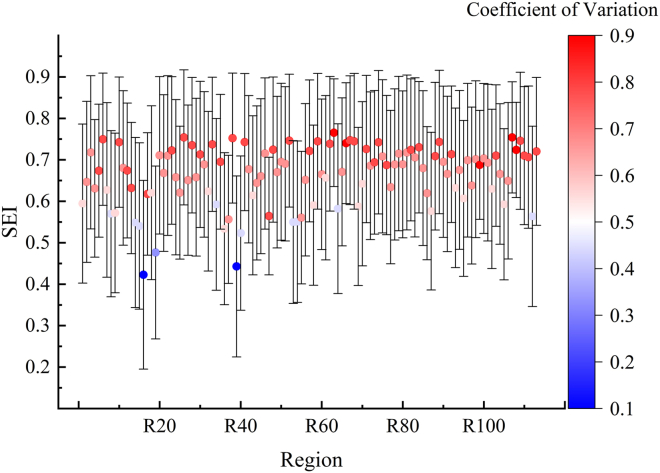
Temporal evolution and coefficient of variation of the SEI across regions in the Fenwei Plain Points indicate the mean SEI of each district-county unit across the study years. Error bars indicate standard deviations, and point colors indicate the coefficient of variation.

Figure 5 summarizes the distribution and variability of SEI across the Fenwei Plain. Most units show annual mean SEI values between 0.6 and 0.8, indicating generally moderate-to-high synergy, while marked spatial heterogeneity remains. Several counties (R20, R40, and R60) exhibit low means but high interannual variability, suggesting fragile synergy with substantial temporal instability. In contrast, other units combine high means with low variability, indicating more stable co-evolution. Dot color represents the coefficient of variation (CV), highlighting a high-synergy-high-volatility subset in which apparently strong synergy is accompanied by limited stability and therefore, merits closer attention.

Table 2 further reports the 2013–2023 change in mean SEI and the relative contributions of its two components. Mean SEI increased from 0.4635 to 0.7503. This improvement is dominated by the coordination component (normalized CCD; +129.6%, 95% CI 119.9–140.2), whereas the alignment component (1 − normalized DTW) contributes negatively (−29.6%, 95% CI −40.2 to −19.9). Together, these results indicate strengthened system-level coordination but persistently lagging temporal alignment, consistent with the residual phase offsets identified by the DTW-based alignment analysis and the spatial mismatch patterns described in the study-area and data overview.

**Table 2:** SEI alignment-coordination structure (2013–2023)

Metric	2013	2023	Value	95% CI
Mean SEI	0.4635	0.7503	+0.2868	—
Alignment	—	—	−29.6	−40.2 to −19.9
Coordination	—	—	129.6	119.9 to 140.2

For mean SEI, the reported value indicates the absolute change from 2013 to 2023, whereas for alignment and coordination, it indicates relative influence (%) on the 2013–2023 change in mean SEI. SEI, synergistic evolution index; CI, confidence interval.

To standardize interpretation, SEI was classified into ten ordered coordination levels over the [0, 1] range, as shown in Table 3. This ten-level scheme provides a consistent basis for describing the intensity and trajectory of carbon-pollution synergy in the subsequent causal modeling and spatial analysis.

**Table 3:** Classification scheme of coordination levels based on the SEI

Level ID	SEI range	Coordination level	Level description
1	(0.0, 0.1]	extremely maladjusted	the system is severely fractured and lacks any meaningful coordination
2	(0.1, 0.2]	severely maladjusted	very low coordination, with clear systemic imbalance
3	(0.2, 0.3]	moderately maladjusted	weak synergy, with consistent signs of non-coordination
4	(0.3, 0.4]	slightly maladjusted	partial misalignment, though some weak synergy may occur intermittently
5	(0.4, 0.5]	marginally maladjusted	near the margin of coordination, with high fluctuation
6	(0.5, 0.6]	barely coordinated	basic synergy is beginning to emerge, though mechanisms need reinforcement
7	(0.6, 0.7]	primarily coordinated	initial coordination mechanisms are in place, but are still limited in extent
8	(0.7, 0.8]	moderately coordinated	noticeable synergistic evolution, with carbon–pollution patterns converging
9	(0.8, 0.9]	well-coordinated	strong synergy effects, with efficient system-level interactions
10	(0.9, 1]	highly coordinated	optimal synergy, with advanced integration of carbon and pollution governance

**Figure 6:**
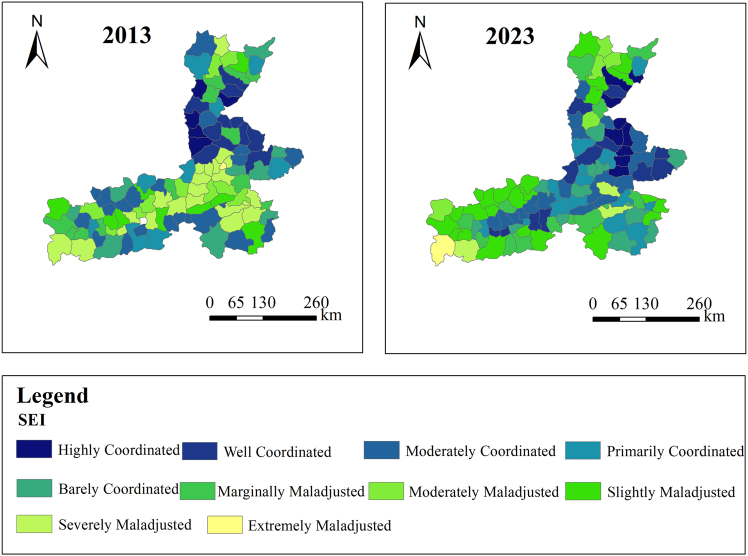
Spatial evolution of carbon-pollution synergy in the Fenwei Plain Scale bars indicate distance in kilometers.

For spatial benchmarking, 2013 and 2023 were used as reference years to highlight the endpoint contrast in SEI levels (Figure 6). Overall, SEI exhibits an upward shift in many units, together with a redistribution of synergy levels across the region. In 2013, the spatial pattern was dominated by maladjusted and barely coordinated classes, whereas highly coordinated units were limited and spatially fragmented. By 2023, well-coordinated and highly coordinated classes expanded along major urban and industrial corridors, indicating the emergence of a more continuous coordination structure between the Fenhe Valley and the Weihe Basin. To demonstrate the dynamic evolution between these endpoints, intermediate-year SEI maps for 2015, 2018, and 2020 are provided in Figure S2, which corroborate that the observed shift is gradual and spatially heterogeneous rather than a two-point artifact.

### Spatial autocorrelation of SEI

#### Global Moran’s I and distance decay

**Figure 7:**
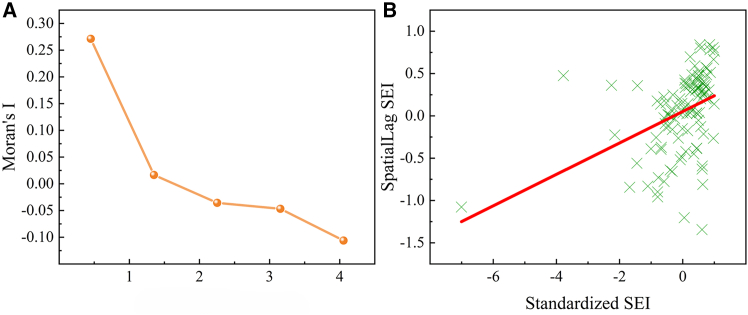
Global spatial autocorrelation analysis of the SEI (A) Moran’s I distance decay curve. (B) Moran’s I scatterplot.

Spatial dependence was assessed using a row-standardized K-nearest-neighbors (KNN) matrix (K = 4) with two-sided permutation tests. At the first-order neighborhood, SEI shows positive global autocorrelation (Moran’s I = 0.27, *p* < 0.01), consistent with clustered high-high and low-low patterns. Moran’s I decreases rapidly with neighborhood order and approaches zero beyond the third order, indicating a short spatial influence range and predominantly local spillovers. These results are shown by the distance-decay curve in Figure 7A and the Moran scatterplot in Figure 7B, in which most observations fall in the first and third quadrants. These local patterns are reported as descriptive spatial evidence and are used to support the interpretation of the response-regime zoning.

#### Local Moran’s I (LISA) and multiple-testing control

**Figure 8:**
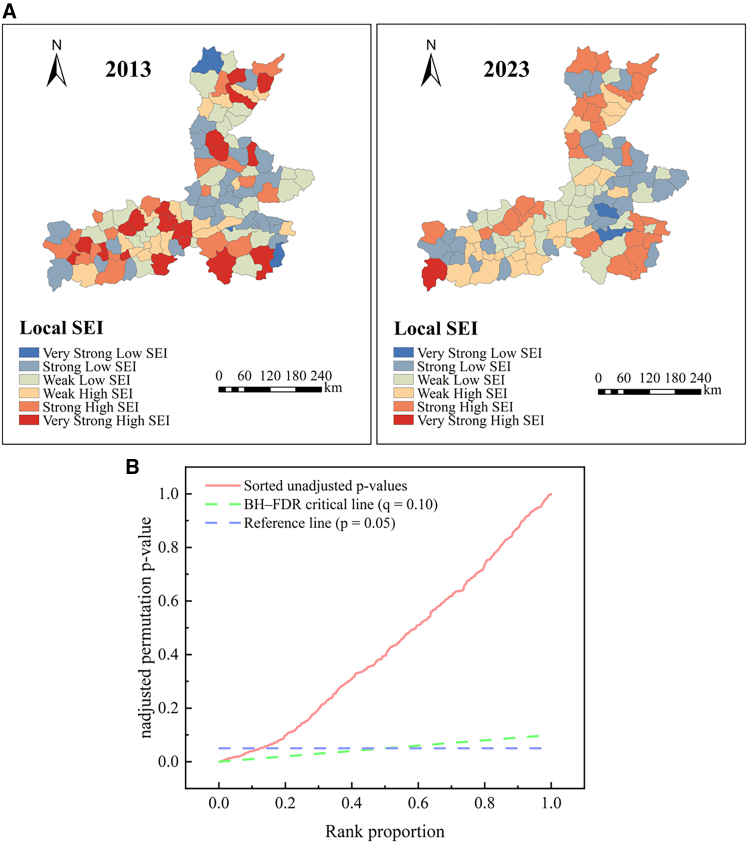
Local spatial autocorrelation of SEI based on Local Moran’s I (A) Descriptive local SEI patterns in 2013 and 2023. Scale bars indicate distance in kilometers. (B) BH-FDR diagnostic plot for local permutation *p* values. *p* values are two-sided local permutation *p* values; dashed lines indicate the Benjamini-Hochberg false discovery rate critical line at q = 0.10 and the nominal reference line at *p* = 0.05.

Local spatial autocorrelation of SEI was further examined using Local Moran’s I. Figure 8A summarizes the local pattern as a descriptive “local SEI” map, where the sign and magnitude of the local statistic are translated into six intensity categories ranging from very strong low to very strong high local SEI. The mapped results indicate a clear temporal adjustment in local spatial structure between 2013 and 2023. In 2013, higher-intensity local patterns were distributed in a more patchy manner across multiple subregions. By 2023, the local structure became more corridor-oriented, with stronger local contrasts emerging along major urban and industrial belts, while several low-intensity areas remained in the peripheral and topographically constrained zones. Intermediate-year local SEI maps for 2015, 2018, and 2020 are provided in Figure S3 to present the full temporal evolution of the local spatial structure.

Local significance was assessed using two-sided permutation tests for each spatial unit. Figure 8B provides a multiple-testing diagnostic by plotting the sorted unadjusted permutation *p* values against the Benjamini-Hochberg false discovery rate (BH-FDR) critical line at q = 0.10, together with a nominal reference line at *p* = 0.05. Under BH-FDR control, the local patterns shown in Figure 8B should be interpreted as descriptive spatial structure rather than statistically confirmed hotspots at the adopted spatial weights specification. This result is consistent with the significant global spatial clustering indicated by the Global Moran’s I analysis, while suggesting that local dependence is sensitive to multiplicity control.

### Bayesian causal mechanism identification

#### Bayesian causal pathway analysis

**Figure 9:**
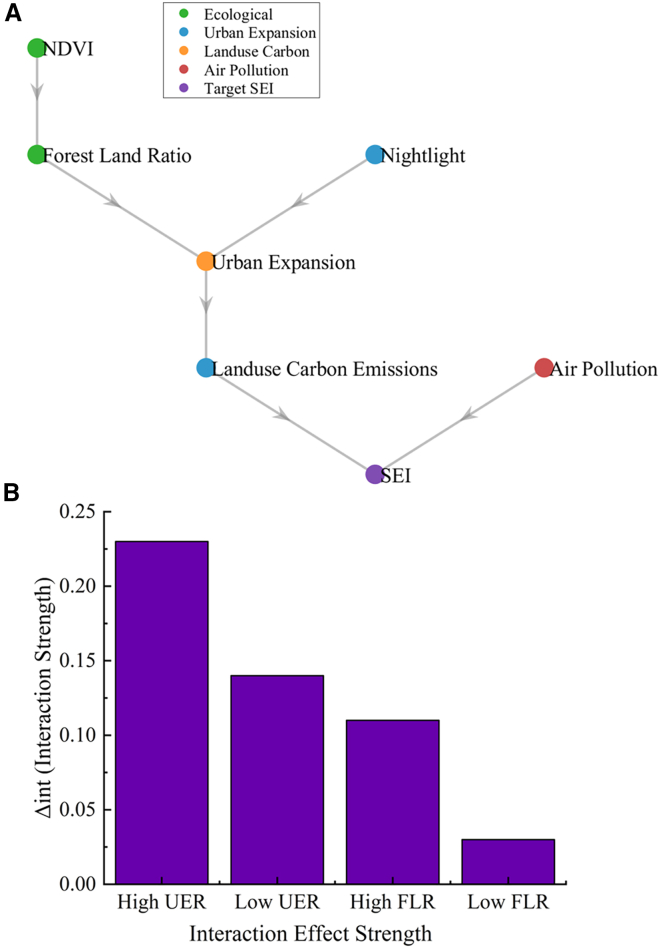
Bayesian causal structure and interaction strength influencing SEI (A) Bayesian network structure with ecological, urban, and emission pathways. (B) Interaction strength of compound variables affecting SEI.

A BN was constructed to identify directional dependencies among key variables relevant to carbon-pollution co-evolution. As shown in Figure 9A, the directed acyclic graph (DAG) places ecological factors, including normalized difference vegetation index (NDVI) and forest land ratio, as root nodes. Urban expansion acts as a central mediator linking ecological conditions to both land-use carbon emissions and NTL intensity, which subsequently influence SEI. To represent potential nonlinear synergies, three interaction terms were included, namely carbon-pollution (C × A), carbon-urban expansion (C × UER), and pollution-forest land (A × FLR). These interaction variables were defined as multiplicative products and discretized into tertiles for interpretability. To maintain causal validity, interaction nodes were connected only to SEI.

Figure 9B indicates that C × UER has the strongest influence on SEI, followed by A × FLR, emphasizing the roles of urban expansion dynamics and ecological buffering. The prominence of C × UER under high UER conditions suggests that rapid urban expansion can intensify the coupled carbon-pollution process, making coordination outcomes more sensitive to joint management. This pattern is consistent with evidence that integrated climate and air quality strategies can yield larger and faster gains than single-pollutant approaches in urban systems where land use and energy demand co-evolve.^46^ National-scale analyses further report that synchronized pathways to carbon neutrality and clean air generate greater co-benefits than piecemeal measures.^47^ Building on these pathways, Table 4 reports interventional SEI distributions under do-operations on carbon and air pollution to quantify the magnitude and direction of policy-relevant effects.

**Table 4:** Interventional distributions of SEI under do-operations on carbon and air pollution

Scenario	E[S]	Pr (S≥3)	Pr (S = 1)	Pr (S = 2)	Pr (S = 3)	Pr (S = 4)
Baseline P(S)	3.219	0.748	0.125	0.127	0.151	0.596
Do (C: high)	3.262	0.754	0.123	0.123	0.123	0.631
Do (A: high)	3.173	0.715	0.129	0.156	0.129	0.587
Do (C: high, A: high)	3.571	0.857	0.071	0.071	0.071	0.786

C denotes land-use carbon emissions and A denotes air pollution. S denotes the discretized SEI level. E[S] is the expected SEI level, and Pr indicates probability under the corresponding do-operation.

Table 4 contrasts the baseline distribution with three high-pressure settings. Relative to baseline, increasing carbon alone yields only a marginal rightward shift (E[S] = 3.262; Pr(S ≥3) = 0.754), whereas increasing air pollution alone slightly reduces the share of higher SEI levels (E[S] = 3.173; Pr(S ≥3) = 0.715). In contrast, the combined intervention concentrates probability mass at S = 4 (0.786) and produces the highest expected level (E[S] = 3.571). Together with the BN pathways, these results suggest complementary influences of carbon and pollution on coordination: single-factor perturbations have small and mixed effects, whereas simultaneous pressure produces a stronger response consistent with the interaction channels in Figure 9B.

To assess robustness to count-based estimation and Laplace smoothing, interventional calculations were repeated under increasing α. As shown in Table S2, the carbon-axis high-to-low total effect declines monotonically with α, but the scenario ordering remains unchanged. The combined intervention consistently dominates the single-factor interventions, whose incremental effects remain small and mixed in direction. This qualitative invariance indicates that the main conclusions are not sensitive to the strength of prior regularization.

#### Bayesian network-based conditional inference and intervention assessment

Conditional SEI distributions under different combinations of land-use carbon emissions (C) and air pollution (A) were further inferred from the BN. SEI was discretized into four levels representing coordination degree. As shown in Figure S4, the prior distribution is slightly right-skewed, indicating an overall tendency toward higher coordination. Under the low-carbon and low-pollution setting (C = 1, A = 1), probability mass mainly concentrates at level 3, whereas the high-carbon and high-pollution setting (C = 4, A =4) shifts the distribution toward level 4. This pattern suggests that stronger joint carbon-pollution pressure is associated with a higher probability of stronger coordination outcomes, rather than a simple linear deterioration of synergy.

Interventional SEI distributions under do-operations on carbon emissions and air pollution further support this interpretation. As reported in Table 4, increasing carbon alone yields only a marginal rightward shift, whereas increasing air pollution alone slightly reduces the share of higher SEI levels. In contrast, the combined high-carbon and high-pollution intervention produces the largest rightward shift, with the highest expected SEI level (E[S] = 3.571) and the highest probability of reaching basically coordinated or higher states (Pr(S ≥3) = 0.857). The probability mass is also concentrated at S = 4, indicating that single-factor perturbations have limited and mixed effects, while simultaneous carbon-pollution pressure activates stronger interaction channels in the BN structure.

**Figure 10:**
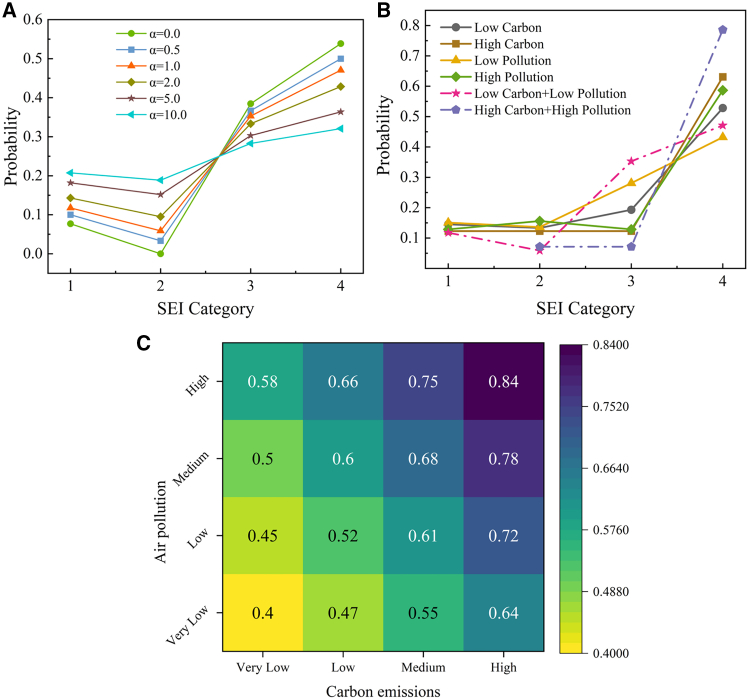
Scenario-based inference and robustness testing of the Bayesian network (A) Sensitivity analysis of the smoothing parameter. (B) Conditional probability variations of SEI under different intervention scenarios. (C) Joint effect of air pollution and carbon emissions on SEI probability.

Robustness and pathway interpretation were further evaluated using scenario contrasts and sensitivity to Laplace smoothing. Figure 10A shows that increasing the smoothing parameter α from 0 to 2 slightly attenuates the probability of higher SEI levels, while the overall distributional pattern remains similar; even when α exceeds 5, higher-SEI states remain predominant. Figure 10B reports conditional inference under different combinations of carbon emissions and air pollution. Under the high-carbon and high-pollution setting, Pr(S = 4) exceeds 0.75, which is higher than both the baseline and single-factor settings. In contrast, under the low-carbon and low-pollution setting, the distribution is more concentrated around level 3 with lower variance. Figure 10C further presents the 4 × 4 matrix across joint C-A combinations, showing an increasing probability of high-SEI outcomes along both axes and broadly similar marginal patterns for carbon emissions and air pollution.

The sensitivity results confirm that the main conclusions are not driven by prior regularization. Although the estimated carbon-axis total effect decreases as the smoothing parameter increases, the scenario ordering remains unchanged, with the combined intervention consistently producing the strongest shift toward higher SEI levels. Specifically, the carbon-axis total effect declines from 0.431 at α = 0 to 0.060 at α = 5, but the qualitative interpretation remains invariant. Detailed sensitivity results are reported in Table S2.

### Spatial clustering and response regimes

#### Synergistic evolution levels and spatial distribution across governance zones

Under the adopted DBSCAN setting in the SEI feature space, the resulting zoning shows clear inter-zone differences in SEI, and one-way ANOVA confirms that between-zone variation is statistically significant (*p* < 0.001). For comparability, K-means was applied to the same SEI feature with the number of clusters fixed at four, and clustering quality was summarized using the silhouette and Davies-Bouldin indices. According to Table 5, the study area is partitioned into four governance zones with distinct gradients in synergy level and stability.

**Table 5:** Classification of synergistic governance zones

Zone name	Explanation
Zone 1	highly coordinated zone; the synergistic governance mechanism is mature and operates with high stability; these areas can serve as regional demonstration units for coordinated carbon and pollution control
Zone 2	moderately to highly coordinated zone; these areas exhibit a relatively high level of coordination with minimal internal variation, making them suitable for the promotion of standardized governance models
Zone 3	basically coordinated zone; although the overall level of coordination is average, internal consistency is high; these regions have the potential for rapid improvement through unified policy implementation
Zone 4	moderately to severely uncoordinated zone; these zones are characterized by significant internal heterogeneity, indicating that customized and adaptive governance strategies are required

Governance zones were identified using DBSCAN clustering based on SEI profiles.

**Figure 11:**
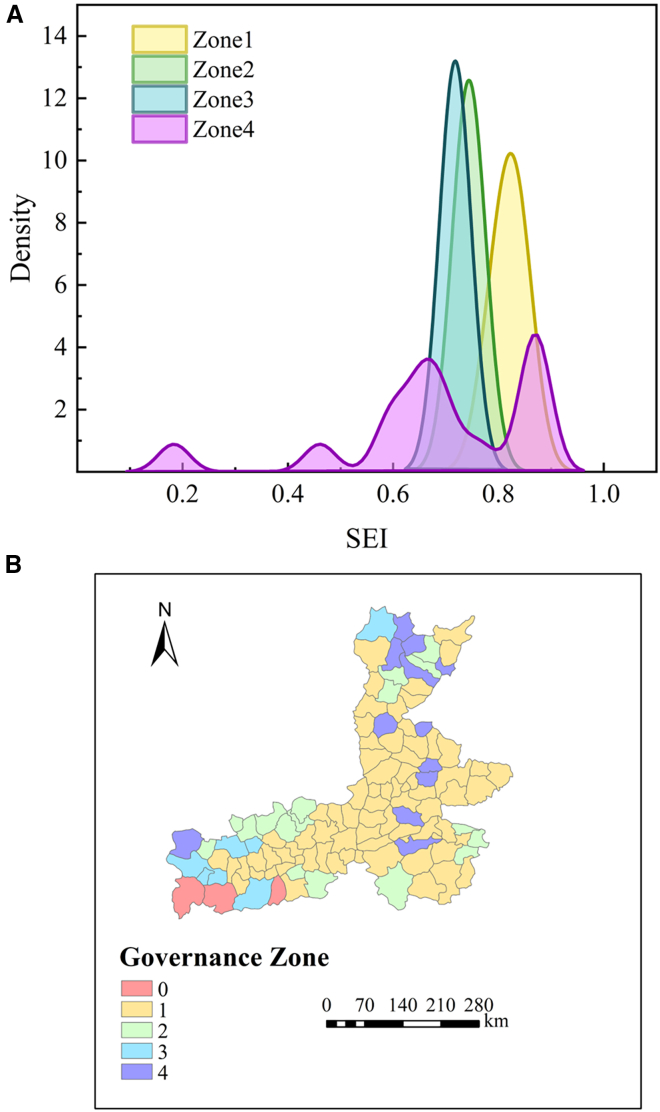
Results of response regimes and SEI distribution characteristics (A) SEI density distribution across governance zones. (B) Spatial distribution of response regimes in the study area. Scale bars indicate distance in kilometers. Zone 0 denotes residual/noise units retained for full spatial coverage and is not treated as an additional governance zone in the four-zone summary.

As shown in Figure 11A, zone 1 concentrates along core urban corridors, including Taiyuan-Jinzhong and Xi’an-Xianyang, combining high SEI with sharply peaked kernel densities, consistent with a stable co-evolution pattern. Zone 2 exhibits relatively high SEI with low dispersion, whereas zone 3 is distributed on the periphery of core urban areas and shows moderate SEI with comparatively consistent internal variation. Zone 2 and zone 3 occupy the transitional and peripheral parts surrounding the core corridors, reflecting intermediate synergy levels with relatively consistent internal distributions. By contrast, zone 4 spans resource-dependent belts in southwestern Shanxi and northern Shaanxi, and its multimodal density and pronounced heterogeneity are consistent with comparatively unstable synergy conditions. Notably, the spatial partition is cross-provincial. Zone 1 is concentrated along the Taiyuan-Jinzhong corridor in Shanxi and the Xi’an-Xianyang corridor in Shaanxi, whereas zone 4 is mainly distributed in resource-dependent belts across southwestern Shanxi and northern Shaanxi, as shown in Figure 11B.

#### ANOVA testing and correlation analysis of key driving factors

One-way ANOVA indicates substantial inter-zone differences in SEI (*p* < 0.001), as shown in Table 6, supporting the statistical separability and interpretability of the four-zone partition. To further examine the structural associations underlying these zoning results, a correlation-based chord diagram was constructed among the key variables and is provided in Figure S5. The results show that urban expansion rate is prominently linked to land-use CO_2_ through Nightlight, while CO_2_ and PM_2.5_ also exhibit a strong association. Ecological indicators, including NDVI and forest land ratio, show beneficial but relatively weaker connections under prevailing urbanization pressure, suggesting that ecological buffering should be combined with demand-side management and structural emission-control measures.

**Table 6:** ANOVA test results for governance zone differences

ZoneID	Zone name	MeanSEI	StdSEI	Explanation
1	zone 1	0.8201	0.0239	the highest mean with low variability
2	zone 2	0.7439	0.0104	moderately high mean with extremely low variability
3	zone 3	0.7180	0.0041	moderate mean with the smallest variability
4	zone 4	0.6869	0.1886	the lowest mean with the highest variability

MeanSEI and StdSEI indicate the mean and standard deviation, respectively, of SEI within each governance zone. One-way ANOVA indicates significant inter-zone differences in SEI, *p* < 0.001.

Collectively, the ANOVA-confirmed inter-zone differences and the supplementary correlation evidence provide an interpretable basis for viewing the four clusters as distinct response regimes. Zone 1 is suited to deep building and industrial retrofits, transport electrification, canopy conservation, and tightened CO_2_-intensity controls per unit of building land, serving as a demonstration cohort for regional co-governance. Zone 2 would benefit from urban growth boundaries and brownfield redevelopment, accelerated fuel switching in small- and medium-sized industries, stricter enforcement of building energy codes, and integrated public transport, with explicit annual targets for land expansion and CO_2_ intensity. Zone 3 requires strengthened ecological buffers, such as near-natural afforestation and riparian restoration, together with guidance toward compact corridor-based growth and cross-jurisdictional coordination on air quality and energy benchmarks. Zone 4 should prioritize plant-level abatement in heavy industry with just-transition support, restoration of degraded land, dual controls on land use and emissions in industrial parks, and the removal of inefficient lighting and underutilized infrastructure that inflates NTLs without commensurate economic output.

The identified response regimes provide empirical evidence consistent with prior assessments emphasizing the co-benefits of integrated carbon and air pollution mitigation, particularly in urban and industrial corridors.^48^^,^^49^

## Discussion

### From static coordination to dynamic co-evolution: Extending the temporal pathway for carbon-pollution synergy

Evidence from the Fenwei Plain indicates that coordination between land-use carbon emissions and air pollution is dynamic rather than static. By integrating temporal alignment (DTW) and structural coordination (CCD) into SEI, synergy is framed as a process shaped by feedbacks and policy adjustment, rather than a single-year snapshot.^50^^,^^51^ This perspective aligns with recent work emphasizing trajectories and processes when assessing joint climate-air outcomes.^52^ In this study, the dynamic property is supported by multi-period observations in 2013, 2015, 2018, 2020, and 2023. Potential phase lags between CO_2_ and PM_2.5_ are accommodated through temporal alignment rather than inferred from two-year snapshots.

### Spatial disparities in synergistic evolution

Despite sustained gains in the regional mean SEI, spatial polarization intensified, with high and stable coordination clustered along core urban corridors, whereas peripheral and topographically constrained areas remained volatile. This divergence is consistent with multi-regional evidence that synergy consolidates where industrial restructuring and energy upgrading advance in tandem but remains fragile where limited local capacity and resources constrain implementation.^53^ The coexistence of high-synergy and high-stability regimes with low-synergy and low-stability regimes indicates that co-governance benefits are unevenly distributed. It also implies that identical policy instruments may yield heterogeneous outcomes when local constraints differ.

### Causality-informed and spatially targeted governance

BN results identify urban expansion and activity intensity, proxied by NTL, as key pathways linking land development to land-use-related CO_2_ and SEI. Structural associations from the chord diagram and dependence patterns inferred by the BN consistently indicate that anthropogenic activity intensity, proxied by NTL, is closely connected to land development dynamics such as urban expansion and to land-use-related CO_2_. At the same time, CO_2_ and PM_2.5_ remain tightly coupled in the inferred structure. These findings suggest that SEI is shaped primarily by the coupling among development intensity, emission intensity, and exposure pressure rather than by any single factor in isolation. Ecological indicators such as NDVI and forest land ratio show beneficial roles, but their links appear comparatively weaker under prevailing urbanization pressures. Ecological buffering is, therefore, more likely to be effective when complemented by demand-side management and structural measures that directly reduce development-driven emission pressure. This mechanism-oriented understanding supports targeted governance that simultaneously manages the pace and intensity of land development while reducing emission intensity rather than relying on ecological interventions alone.

The results indicate that coordination tends to strengthen when both emission intensity and pollution pressure are high, which is consistent with the activation of feedback mechanisms under higher stress levels.^54^^,^^55^ This interpretation is also consistent with prior evidence showing that urban spatial growth and activity management condition the effectiveness of integrated climate-air policies.^56^^,^^57^

### Comparative perspective on climate-air co-governance

International assessments conclude that jointly designed greenhouse gas and air quality measures deliver durable co-benefits and spatially coherent improvements, particularly in urban and industrial belts. Model-based studies report substantial reductions in fine particulate exposure under climate policy packages in Europe and marked local health gains from accelerated carbon mitigation in the United States.^58^ Within China, synergy tends to strengthen where industrial upgrading and energy restructuring advance rapidly, and clustered co-benefits have been documented since the rollout of combustion-focused air quality controls.^59^^,^^60^ This evidence base is consistent with the Fenwei Plain, where corridor-oriented coordination emerges alongside pronounced heterogeneity across resource-dependent belts.^61^ Such a pattern reinforces the need for place-based co-governance that explicitly accounts for spatial constraints.

The corridor-oriented coordination observed in the Fenwei Plain also reflects a broader governance challenge faced by regional systems undergoing land-use transition, industrial restructuring, and air quality improvement. In these contexts, land-use-related carbon emissions and PM_2.5_ pollution may not respond synchronously to policy interventions or structural change. The DTW-CCD-based SEI provides a transferable diagnostic metric for distinguishing stable co-mitigation from apparent coordination driven by short-term fluctuations or lagged responses. This feature makes the framework relevant to other corridor-based urban agglomerations, resource-dependent regions, industrial transition zones, and rapidly urbanizing inland areas where land expansion, ecological constraints, emission growth, and pollution reduction interact unevenly across space.

The transferability of the framework should be understood as methodological rather than region-specific. The analytical pathway from dynamic synergy measurement to Bayesian pathway diagnosis and spatial response-regime identification can be adapted to comparable carbon-pollution co-governance contexts. However, the specific zoning pattern identified in the Fenwei Plain should not be directly transplanted to other regions. Explanatory variables, SEI classification thresholds, BN structures, spatial-weight settings, and DBSCAN parameters need to be recalibrated according to local land-use categories, pollution-source profiles, data availability, and governance objectives. With such local calibration, the proposed framework can support place-sensitive co-governance assessment while preserving the specificity of regional policy interpretation.

### Optimizing response regimes

The four-zone response regime derived from DBSCAN and validated by ANOVA provides a tractable blueprint for differentiated action, including consolidation in high-stability areas, standardized promotion where dispersion is low, transitional guidance where improvement potential is evident, and compensatory support where heterogeneity is pronounced. This zoning is empirically supported by the statistically significant inter-zone differences and the spatially coherent partitions reported in the governance-zoning results. This architecture resonates with insights from polycentric and multi-level governance, but extends them by explicitly anchoring zoning in SEI dynamics and the mechanism-relevant linkages identified by the BN and the chord-based association structure.^62^^,^^63^ In practice, the regime-based design helps avoid one-size-fits-all prescriptions by matching policy packages to dominant constraints and stability profiles across zones.

### Policy implications

The findings highlight the need for spatially adaptive governance strategies to address heterogeneous carbon-pollution coordination across the Fenwei Plain. Accordingly, a zoning-based mitigation approach is recommended to match policy instruments to local coordination characteristics and development constraints. In high- and stable-synergy areas, priorities include consolidating gains through industrial retrofitting, energy-efficient building renovations, and tighter carbon-intensity caps per unit of building land, supported by coordinated transport electrification and industrial heat decarbonization. In moderately synergistic but spatially homogeneous areas, standardized measures such as urban growth boundaries, brownfield redevelopment, building energy codes, and fuel switching in medium-sized industries can help sustain co-benefit trajectories. Areas with intermediate synergy and higher improvement potential should emphasize ecological restoration, compact urban development, and multimodal transport expansion alongside institutionalized cross-jurisdictional air pollution coordination. In lagging and heterogeneous counties, compensatory support is needed, including targeted emission reductions in high-emitting industries, dual controls on industrial-park expansion and emissions, and ecological compensation and performance-based fiscal transfers; inefficient infrastructure that inflates proxy indicators without substantive output should also be phased out. To operationalize differentiated policies, a compact indicator set is proposed, including carbon intensity per unit of building land, annual PM_2.5_ levels, SEI stability, and NTL dynamics, to support performance contracts, fiscal arrangements, and regional benchmarking.

Provincial differentiation is required because the Fenwei Plain spans central and southern Shanxi, the Guanzhong region of Shaanxi, and western Henan. Along the Shanxi corridor, including the Taiyuan-Jinzhong belt, the priority is to consolidate synergy gains through industrial and energy restructuring and strict CO_2_ intensity control in mature governance regimes. In the Guanzhong corridor, including the Xi’an-Xianyang belt, emphasis should be placed on managing development intensity and urban spatial form to prevent carbon rebounds while sustaining PM_2.5_ improvements. In western Henan, where cross-boundary transport and mixed sources are prominent, strengthened inter-jurisdictional coordination and unified monitoring are needed to prevent local rebounds from offsetting regional co-benefits.

### Uncertainty, sensitivity, and limitations

This study involves uncertainty from both data inputs and model specifications. At the data level, land-use-based CO_2_ accounting is influenced by land-use classification accuracy and the selection of emission coefficients. Gridded PM_2.5_ products and district-county aggregation may further smooth fine-scale variability. Accordingly, results are interpreted as comparable evidence for spatial and temporal contrasts under a consistent processing pipeline rather than deterministic predictions of absolute levels at finer scales.

At the method level, uncertainty may accumulate across sequential modules. DTW-based alignment depends on observed time series and, therefore, reflects sample-specific temporal variability. SEI integrates alignment and coordination components and may vary with aggregation settings. Robustness was assessed through sensitivity analysis of SEI weights and resampling-based uncertainty quantification, with key summaries reported together with uncertainty bounds.

BN results are subject to uncertainty from structure learning and estimation with discretized variables. Structural stability was evaluated via bootstrap resampling, and the robustness of interventional results was examined under alternative Laplace smoothing settings. Although effect magnitudes attenuated as smoothing increased, the qualitative ordering of intervention scenarios remained stable. Zoning results from density-based clustering depend on neighborhood parameterization and the treatment of noise points. Here, zoning was implemented using the baseline neighborhood settings specified in the STAR Methods, noise points were retained as a residual group to ensure full spatial coverage, and inter-zone separation was supported by ANOVA. Sensitivity settings and robustness summaries are consolidated in the supplemental information (Table S1; Figure S6).

Several limitations should be acknowledged. First, the framework focuses on key indicators and does not explicitly represent sectoral emissions or atmospheric chemistry. Second, while BNs provide interpretable dependence structures and interventional contrasts, causal interpretation is conditional on the included variables and modeling assumptions. Third, governance zones should be interpreted as data-driven response regimes rather than fixed administrative categories, and implementation requires alignment with local planning and regulatory contexts.

Integrating the preceding results, our multi-stage framework highlights that carbon-pollution synergy in the Fenwei Plain improved from 2013 to 2023, yet spatial disparities persisted. High-synergy and stable units became increasingly concentrated along the Fenhe-Weihe urban belt, whereas lower-synergy and less stable units remained in ecologically fragile or resource-dependent subregions. BN analysis indicates that urban expansion serves as a central pathway linking ecological conditions, NTL intensity, and coordination outcomes, with stronger synergy responses arising under simultaneous high-carbon and high-pollution conditions. Density-based clustering and ANOVA further identify four distinct response regimes, suggesting that effective regional co-mitigation should move beyond uniform policy design toward spatially differentiated governance informed by local coordination status and mechanism pathways. While the framework was developed for the Fenwei Plain, it provides a transferable analytical pathway for diagnosing dynamic carbon-pollution synergy and guiding place-sensitive governance in other corridor-based urban agglomerations, resource-dependent regions, and rapidly urbanizing areas, provided that local calibration of explanatory variables, classification thresholds, spatial settings, and zoning parameters is conducted.

### Limitations of the study

Although the proposed framework provides an interpretable pathway for diagnosing carbon-pollution co-evolution and spatially differentiated response regimes, several limitations remain. The BN analysis identifies probabilistic dependencies and mechanism-relevant pathways among the selected variables, but the inferred relationships should be interpreted as diagnostic evidence rather than definitive causal proof. The results may still be influenced by variable selection, discretization strategy, and the available temporal span of district-county observations. Future work could incorporate longer time series, finer-scale socioeconomic and environmental variables, and additional external validation to further verify the identified causal pathways and strengthen the operational design of differentiated carbon-pollution co-governance strategies.

## Resource availability

### Lead contact

Further information and requests for resources should be directed to and will be fulfilled by the lead contact, Bilin Shao (sblin@xauat.edu.cn).

### Materials availability

This study did not generate new unique reagents or materials.

### Data and code availability

• This study uses existing publicly available datasets. These datasets are listed in the key resources table.
• All code supporting the findings of this study is available at our GitHub repository: https://github.com/mimi-xue/SEI_CausalZoning_Code.
• Any additional information required to reanalyze the data reported in this paper is available from the lead contact upon reasonable request.

## Acknowledgments

This work was supported by the Humanities and Social Sciences Research Base Project of the 10.13039/501100007957Chongqing Municipal Education Commission (no. 25SKJD068), the 10.13039/501100001809National Natural Science Foundation of China (Youth Program; no. 72504216), the 10.13039/501100017596Natural Science Basic Research Program of Shaanxi Province (General Project; no. 2025JC-YBMS-830), the Youth Innovation Team of Shaanxi Universities (no. Z20230765), and the Chongqing Social Science Planning Doctoral Project (no. 2024BS083).

## Author contributions

Conceptualization, B.S.; methodology, X.Z., B.S., J.S., and N.T.; software, X.Z. and N.T.; formal analysis, X.Z.; visualization, X.Z., B.S., and J.S.; data curation, W.Z. and X.L.; validation, W.Z.; writing ‑ original draft, X.Z.; supervision, B.S.

## Declaration of interests

The authors declare no competing interests.

## STAR★Methods

### Key resources table

**Table:** 

REAGENT or RESOURCE	SOURCE	IDENTIFIER
**Deposited data**		

Land-use maps (30 m; 2013–2023)	Resource and Environment Science and Data Center (RESDC), Chinese Academy of Sciences	https://www.resdc.cn/
Administrative boundaries (Fenwei Plain; prefecture and district–county units)	RESDC, Chinese Academy of Sciences	https://www.resdc.cn/
NDVI products	RESDC, Chinese Academy of Sciences	https://www.resdc.cn/
Annual mean PM_2.5_ concentration dataset (2013–2023)	China High Air Pollutants (CHAP) dataset, Tsinghua University	https://weijing-rs.github.io/product.html
Nighttime lights (VIIRS DNB; harmonized series)	Earth Observation Group (EOG), Payne Institute, Colorado School of Mines	https://eogdata.mines.edu/products/vnl/

**Software and algorithms**		

ArcGIS Desktop	ESRI	https://www.esri.com/en-us/arcgis/products/arcgis-desktop/overview
SEI_CausalZoning_Code	This paper	https://github.com/mimi-xue/SEI_CausalZoning_Code
DTW-CCD and SEI calculation scripts	This paper	https://github.com/mimi-xue/SEI_CausalZoning_Code
Bayesian network modeling scripts	This paper	https://github.com/mimi-xue/SEI_CausalZoning_Code
DBSCAN zoning scripts	This paper	https://github.com/mimi-xue/SEI_CausalZoning_Code
Statistical analysis scripts	This paper	https://github.com/mimi-xue/SEI_CausalZoning_Code

### Method details

#### Data sources and preprocessing

Multi-source panel data were compiled for the Fenwei Plain from 2013 to 2023 and harmonized at the district–county scale. Annual 30 m land-use rasters were obtained from the Resource and Environment Science and Data Center (RESDC). Annual fine particulate matter (PM_2.5_) concentrations were obtained from the China High Air Pollutants (CHAP) dataset, and mean values were aggregated to both district–county and prefecture levels. Administrative boundary data were also obtained from RESDC to ensure consistency in spatial aggregation, statistical calculation, and visualization. The Normalized Difference Vegetation Index (NDVI) was obtained from RESDC and used to characterize vegetation conditions. Nighttime lights (NTL), used as a proxy for urban activity intensity and anthropogenic disturbance, were derived from harmonized Visible Infrared Imaging Radiometer Suite (VIIRS) Day and Night Band (DNB) nighttime light products.

To ensure comparability across datasets, all spatial layers were processed using a consistent administrative-unit framework. Land-use data were classified according to the Land Use Classification Standard of China (GB/T 21010–2017) and reclassified into six categories: plowland, woodland, grassland, waters, building land, and unused land. The forest land ratio was calculated as the forested land area divided by the total area of each administrative unit. The urban expansion rate was calculated as the average annual growth rate of building land area based on multi-period land-use maps and was computed for each district–county unit. All variables used for subsequent modeling were extracted at consistent spatial units and standardized using *Z* score transformation before model construction.

#### Land-use carbon emission accounting

In land-use-based carbon accounting, ecological land types contribute to the carbon cycle primarily through sequestration, whereas building land serves as the spatial carrier of anthropogenic activities. Although emissions from building land can be estimated more precisely using energy-consumption data, such data are often unavailable or inconsistent for long-term, multi-regional, and county-level analyses. Therefore, to ensure cross-regional comparability in the Fenwei Plain under data constraints, this study adopts an area-based emission-coefficient approach, which provides a practical estimate of the macro-scale carbon impact associated with urban land expansion. Carbon emissions from land use are calculated as follows:

(Equation 1) $$E_{CO_{2}}=\sum_{i=1}^{n}A_{i}\times \beta_{i}\text{,}$$

where $E_{CO_{2}}$ represents the total carbon emissions from land use in the study area (unit: t, metric tonnes); *A*_*i*_ denotes the area of land-use type *i*(unit: hm^2^); *β*_*i*_ is the carbon emission coefficient for land-use type *i* (unit: t·hm^−2^); *n* is the number of land-use types.

#### Synergistic evolution index construction

##### Dynamic time warping

In this study, Dynamic Time Warping (DTW) is used to quantify the temporal similarity between land-use carbon emissions and PM_2.5_ concentration changes. DTW is a flexible sequence-alignment algorithm that identifies the minimum cumulative distance between two time series by allowing non-linear temporal matching, making it suitable for environmental sequences with phase shifts and heterogeneous fluctuation patterns.^64^ Compared with Euclidean distance, DTW enables elastic time-axis alignment to capture asynchronous evolution and lagged co-movements between land-use carbon emissions and PM_2.5_.^65^ The formal definition of DTW is provided as follows:

(Equation 2) $$DTW\left(X,Y\right)=\min_{\left\{W_{k}\right\}}\left\{\sqrt{\sum_{k=1}^{K}d}{\left(x_{ik},y_{jk}\right)}^{2}\right\}\text{,}$$

where *X* denotes the first time series, *Y* denotes the second time series, *d*(*x*_*i*_,*y*_*j*_) represents the distance between points, *w*_*k*_ denotes the *k* element of the alignment path.

##### Modified coupling coordination degree

To assess the synergistic development between land-use carbon emissions and PM_2.5_ concentrations, a modified coupling coordination degree (CCD) model is applied to DTW-aligned sequences. Entropy weighting is used to derive comprehensive performance scores for each subsystem, which are then used to compute the coupling degree and coordination level. Using standardized data, a weighted composite score is first obtained for each evaluation unit, and the coupling degree is subsequently calculated as follows:

(Equation 3) C=(∏i=1nUi(1n∑i=1nUi)n)1n

where *U*_*i*_ denotes the composite performance score of subsystem *i*. Note that *C* in Equation 3 denotes the coupling degree of the CCD model. The coupling degree reflects the interaction intensity between the two subsystems. To represent the overall development level, the average performance score is computed as follows:

(Equation 4) $$T=\frac{1}{n}\sum_{i=1}^{n}U_{i}$$

By combining the coupling degree *C* and the composite development level *T*, the Coupling Coordination Degree *D* is calculated to represent the coordinated evolution level between the two systems, as defined:

(Equation 5) $$D=\sqrt{C\times T}$$

In this formula, a higher value of *D*∈[0,1] indicates a stronger and more coordinated relationship between the two systems. By jointly accounting for structural interaction (via the coupling degree) and subsystem development level, the modified CCD model alleviates the limitation of traditional coupling approaches that ignore system growth, thereby providing a more informative measure of the synergistic evolution between land-use carbon emissions and air pollution.

##### Construction of the synergetic evolution index

In this study, the Synergistic Evolution Index (SEI) is proposed as a process-sensitive coupling metric by integrating DTW and a modified CCD framework to quantify the synergy between land-use carbon emissions and PM_2.5_ concentrations. DTW captures temporal misalignment and nonlinear co-movement between the two series, thereby characterizing lag-aware co-evolution that static coupling indices cannot represent. In parallel, the CCD component evaluates coordinated development based on normalized subsystem performance and reflects the overall coupling and coordination strength between the two subsystems. By integrating these complementary dimensions, SEI jointly measures lag-aware temporal co-evolution and systemic coordination, providing a unified indicator for diagnosing dynamic carbon–pollution coupling within the regional system.

To ensure comparability, DTW values and coordination scores are normalized to the [0,1] range. Because DTW is negatively oriented (smaller values indicate stronger synergy), inverse normalization is applied as follows:

(Equation 6) $$DTW_{norm}=1-\frac{DTW-\min\left(DTW\right)}{\max\left(DTW\right)-\min\left(DTW\right)}$$

As the coordination degree is positively oriented (higher values indicate better coordination), it is normalized using min–max scaling as defined:

(Equation 7) $$Coord_{norm}=\frac{Coordination-\min\left(Coordination\right)}{\max\left(Coordination\right)-\min\left(Coordination\right)}$$

On this basis, the SEI is constructed as follows, as defined:

(Equation 8) $$SEI=\alpha \cdot DTW_{norm}+\left(1-\alpha \right)\cdot Coord_{norm}$$

where α is the weight assigned to the normalized DTW component, and 1 − α is the weight assigned to the normalized coordination component.

In the baseline specification, the weights were set to *α* = 0.5, assigning equal importance to evolutionary alignment and developmental coordination, which is a common convention in composite index construction when no strong *a priori* evidence favors either component. Robustness was evaluated by varying *α* within (0.3, 0.7). The resulting spatiotemporal SEI patterns and the identification of high-synergy and low-synergy clusters remained qualitatively consistent across weight settings; only minor numerical differences were observed, without altering regime classification or policy-relevant conclusions.

For reporting, SEI was summarized by year and additively decomposed into an Alignment component (1− min–max normalized DTW after 1–99% winsorization) and a Coordination component (min–max normalized CCD). Uncertainty was quantified via a unit-level bootstrap, using administrative units (*n* = 113) as the resampling unit with *B* = 1000 replications, and 95% percentile confidence intervals.

#### Spatial autocorrelation analysis

Spatial autocorrelation was used to quantify statistical dependence among geographic units. Two complementary statistics were employed: Global Moran’s I for areawide association and Local Moran’s I (LISA) for neighborhood-scale heterogeneity. Let *n* denote the number of spatial units, *x*_*i*_ is the attribute value of the spatial unit *i*, $\bar{x}$ is the mean of all attribute values, *ω*_*ij*_ represents the spatial weight, We adopt a row-standardized K-nearest neighbors weight with *K* = 4, setting *ω*_*ii*_ = 0 and *ω*_*ij*_ = 1/*K* when *j* is among the four nearest neighbors of *i*, and *ω*_*ij*_ = 0. Define $S_{0}=\sum_{i=1}^{n}\sum_{j=1}^{n}\omega_{ij}$.The Global Moran’s I is presented as follows:

(Equation 9) $$I=\frac{n}{S_{0}}\cdot \frac{\sum_{i=1}^{n}\sum_{j=1}^{n}\omega_{ij}\left(x_{i}-\bar{x}\right)\left(x_{j}-\bar{x}\right)}{\sum_{i=1}^{n}{\left(x_{i}-\bar{x}\right)}^{2}}$$

Positive and significant *I* indicates positive spatial autocorrelation (similar values cluster), whereas negative and significant *I* indicates spatial dispersion.

For local dependence, local spatial autocorrelation is used to characterize heterogeneity within each unit’s neighborhood. The Local Moran’s I statistic is defined as follows:

(Equation 10) $$I_{i}=\left(x_{i}-\bar{x}\right)\sum_{j=1}^{n}\omega_{ij}\left(x_{j}-\bar{x}\right)$$

In this formula, *I*_*i*_ represents the local spatial autocorrelation index of the *i* spatial unit.

Inference for both global and local statistics uses 999 two-sided randomization tests. For local tests, multiplicity is controlled using the Benjamini–Hochberg false discovery rate with q set to 0.10. Unless otherwise specified, significant results refer to permutation-based *p*-values under these settings. Spatial autocorrelation statistics are used as diagnostic tools to characterize the spatial dependence of SEI and to provide supporting evidence for interpreting the zoning results.

#### Bayesian causal mechanism modeling

This study adopts a Bayesian network (BN) to identify causal relationships between SEI and key driving factors. A BN represents conditional dependencies among variables using a directed acyclic graph (DAG) together with conditional probability distributions, thereby enabling the identification of plausible causal pathways.^66^ Formally, a BN can be denoted as *G*=(*V*,*E*), where *V* denotes the set of variable nodes and *E* represents the set of directed edges. The joint probability distribution can then be factorized according to the DAG structure, as shown:

(Equation 11) $$P\left(X_{1},X_{2},\cdots ,X_{n}\right)=\prod_{i=1}^{n}P\left(X_{i}|Pa\left(X_{i}\right)\right)$$

where *Pa*(*X*_*i*_) denotes the set of parent nodes of the variable *X*_*i*_. The BN structure *G* and its parameters *θ* are estimated by maximizing the posterior probability, as shown:

(Equation 12) P(G,θ|D)∝P(D|G,θ)P(θ|G)P(G)

where *D* denotes the observed dataset, *θ* represents the model parameters, *P*(*D*|*G*,*θ*) is the likelihood function, *P*(*G*) is the network structure, and *P*(*θ*|*G*) represents the prior distribution.

During structure learning, a hill-climbing algorithm is used to search for the optimal network structure under the Bayesian Information Criterion (BIC). The BIC scoring function is defined:

(Equation 13) $$BIC\left(G\right)=\log\;P\left(D|G,\hat{\theta }\right)-\frac{d}{2}\log\left(N\right)$$

where $\hat{\theta }$ denotes the maximum likelihood estimates of the parameters under the given structure *G*, and *d* represents the number of free parameters in the model. Subsequently, the bootstrap resampling method is employed to perform stability validation of the network structure.

#### Spatial clustering and zoning

To characterize spatial heterogeneity in the synergistic evolution of land-use carbon emissions and air pollution, density-based spatial clustering was used to delineate governance zones in the Fenwei Plain. The clustering was performed on SEI-derived features to identify data-driven response regimes, and the resulting zones were subsequently interpreted together with spatial dependence diagnostics and causal pathway evidence. DBSCAN identifies clusters of irregular shape and is robust to noise and outliers.^67^ Let *D* denote the dataset in the SEI feature space, and let *dist*(*p*,*q*) be the Euclidean distance. The neighborhood of any point *p*∈*D* is defined as follows:

(Equation 14) $$N_{\varepsilon }\left(p\right)=\left\{q\in D|dist\left(p,q\right)\leq \varepsilon \right\}$$

A point *p* is a core point if |*N*_*ε*_(*p*)|≥*MinPts*. Clusters are formed by iteratively expanding from core points via density-reachability; points that are neither core nor density-reachable from any core point are labeled noise.

In the baseline implementation, clustering was performed on normalized SEI-derived features. The neighborhood radius was set to *ε* = 0.1, and the minimum number of points was set to MinPts = 8 for the 113 district–county units. Units classified as noise were retained and reported as an additional residual group to ensure full spatial coverage in the zoning interpretation. Cluster separation was assessed using one-way ANOVA on SEI.

For comparison, K-means was applied to the same SEI feature. The number of clusters was set to four to match the governance-zone structure used for response-regime interpretation. Clustering quality was summarized using the silhouette score and the Davies–Bouldin index.

#### Uncertainty and sensitivity assessment

To evaluate the robustness of the integrated framework, uncertainty quantification and sensitivity checks were conducted across key modules. For SEI construction, the component weights were varied from 0.3 to 0.7 around the baseline equal-weight setting. Sampling uncertainty in SEI summaries was quantified using unit-level bootstrap resampling with 1000 replications and 95% percentile confidence intervals. For Bayesian network modeling, bootstrap resampling was used to assess structural stability, and intervention results were evaluated under different Laplace smoothing settings. For DBSCAN zoning, the neighborhood parameters were fixed at ε = 0.1 and MinPts = 8, and noise points were retained as a residual group to ensure full spatial coverage. Additional Bayesian-network inference and robustness results are provided in the Supplemental Information (Tables S1 and S2; Figures S4 and S6).

### Quantification and statistical analysis

All statistical and computational analyses were conducted at the district–county scale, with 113 spatial units in the Fenwei Plain. The core quantitative framework was centered on the construction of the Synergistic Evolution Index (SEI), which integrates Dynamic Time Warping (DTW) and the Coupling Coordination Degree (CCD) to jointly characterize temporal misalignment, lag-aware co-movement, and system-level coordination between land-use carbon emissions and PM_2.5_. Uncertainty in SEI summaries was quantified using unit-level bootstrap resampling (B = 1000) with percentile-based 95% confidence intervals, and sensitivity of the SEI to alternative weight settings was evaluated by varying *α* from 0.3 to 0.7. To support structural interpretation, Bayesian network analysis was used to identify key driver chains, with bootstrap resampling applied to assess network stability and additional sensitivity checks conducted for Laplace smoothing settings. To translate the dynamic SEI patterns into operational governance types, DBSCAN was used to identify response regimes, and inter-zone differences were evaluated using one-way ANOVA. Clustering validity was assessed using the silhouette score and the Davies–Bouldin index, with K-means used as a benchmark method. Spatial dependence was further examined using Global Moran’s I and Local Moran’s I based on 999 two-sided randomization tests. Multiple testing for local statistics was controlled using the Benjamini–Hochberg false discovery rate with q = 0.10. In this framework, spatial autocorrelation analysis was used as a diagnostic and validation tool for the spatial structure of SEI and the interpretability of the zoning results, rather than as the core mechanism-identification module. Spatial data processing was conducted in ArcMap. Additional parameter settings, robustness checks, and supplementary results are provided in the Supplemental Information.
