# Supplementary material for: Unraveling land-use carbon-pollution co-evolution: A dynamic coordination framework with causal pathways and spatial zoning
Source: iScience. 2026 Jun 27;29(7):116520. doi: 10.1016/j.isci.2026.116520 (PMC13378384; doi:10.1016/j.isci.2026.116520)
Supplement: Document S1. Figures S1–S6; Tables S1 and S2 [file mmc1.pdf]

iScience, Volume *29*

Supplemental information

Unraveling land-use carbon-pollution co-evolution: A dynamic coordination framework with causal pathways and spatial zoning

Xue Zhao, Bilin Shao, Jia Su, Ning Tian, Wei Zhao, and Xinyu Liu

Supplemental information

SUPPLEMENTARY FIGURES AND TABLES

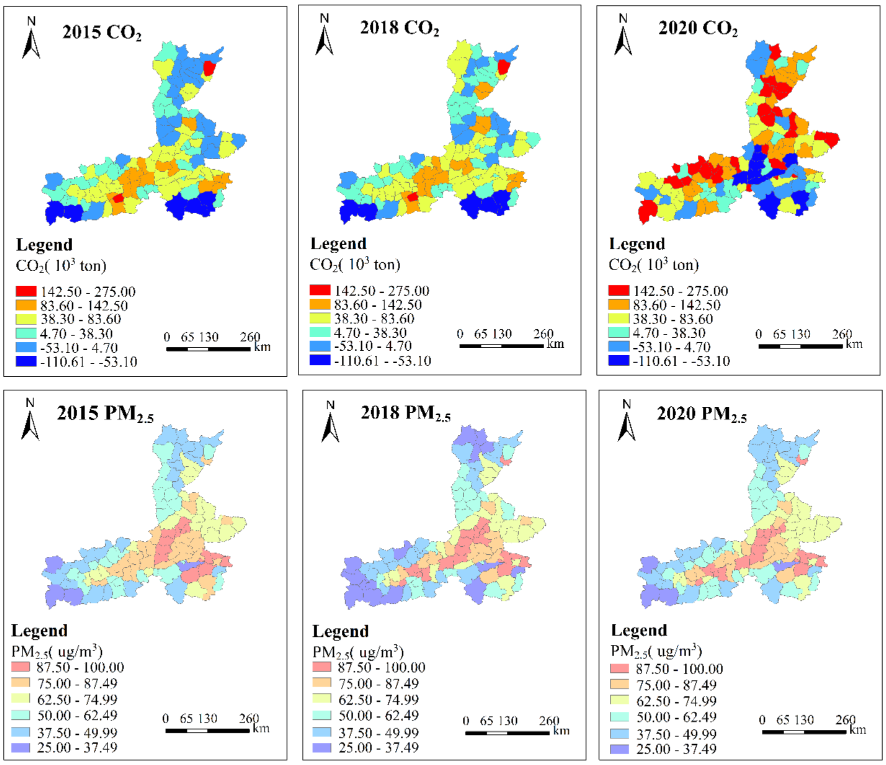

Figure S1. Intermediate-year spatial patterns of land-use-related CO_2 emissions and $PM_{2.5}$ concentrations in the Fenwei Plain, Related to Figure 2.

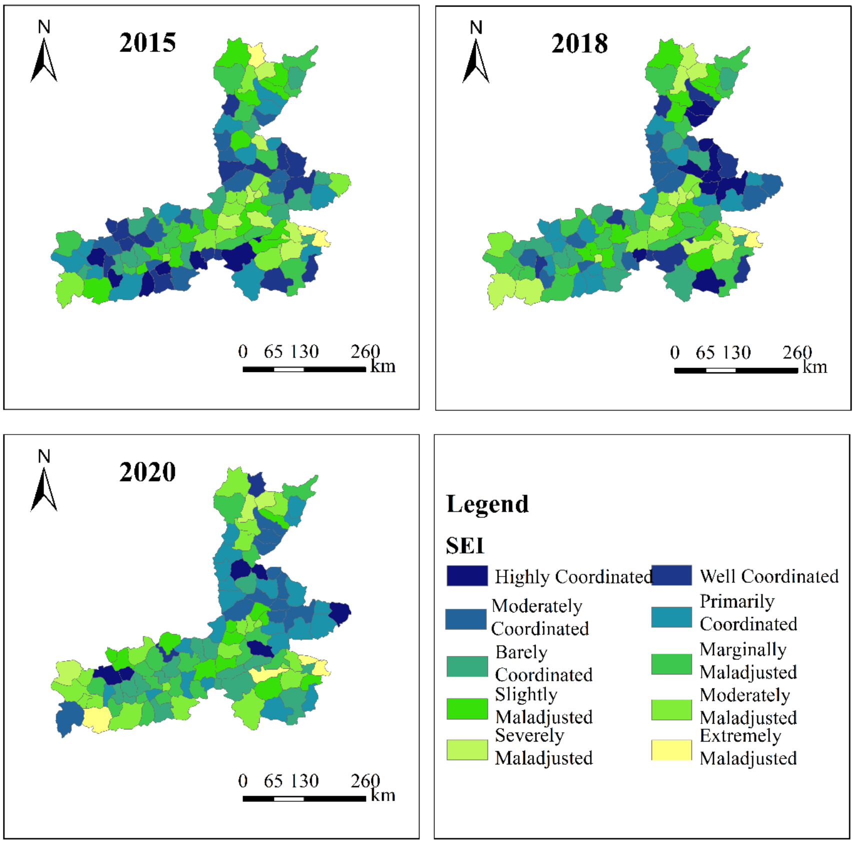

Figure S2. Intermediate-year spatial distribution of SEI levels in the Fenwei Plain, Related to Figure 6.

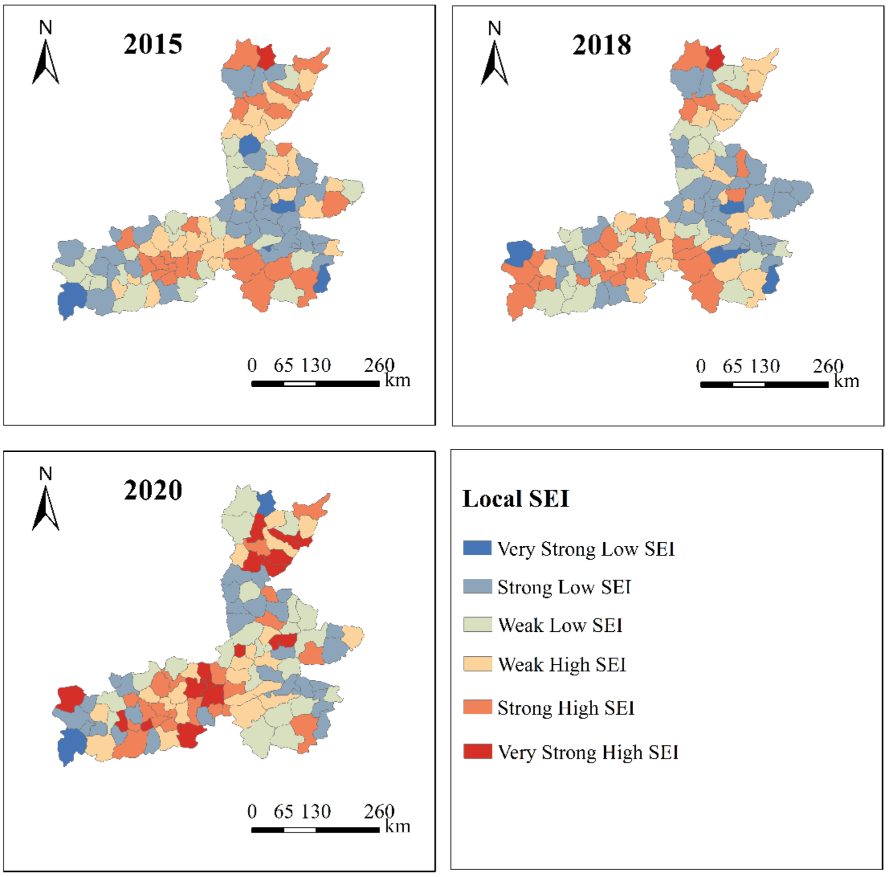

Figure S3. Intermediate-year Local SEI patterns based on Local Moran's I in the Fenwei Plain, Related to Figure 8.

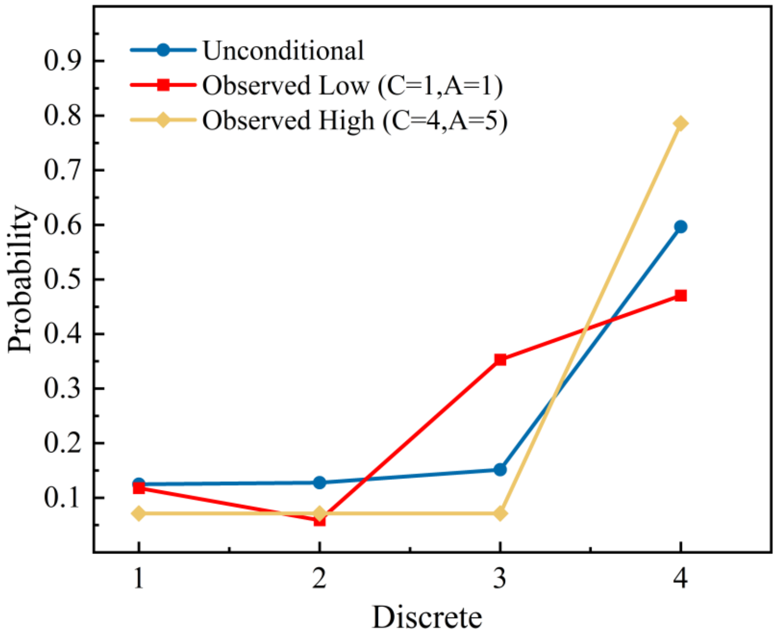

Figure S4. Conditional probability distribution curves of SEI under the BN model, Related to Figure 10.

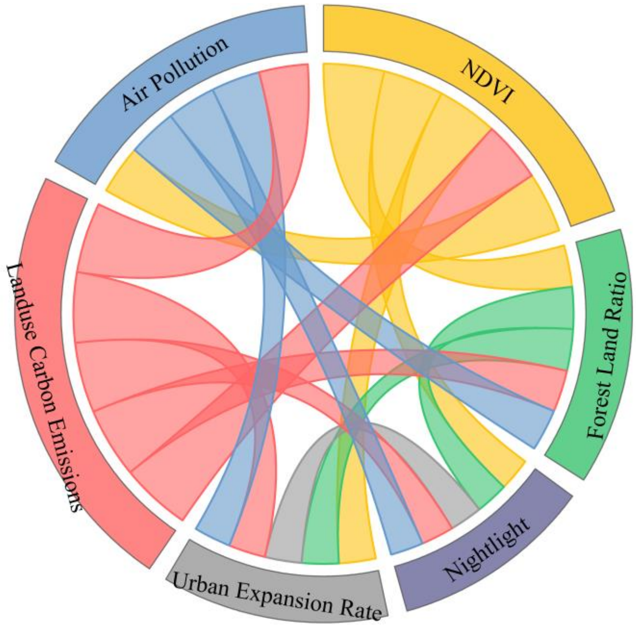

Figure S5. Coupling structure of multivariate synergistic relationships, Related to Figure 11.

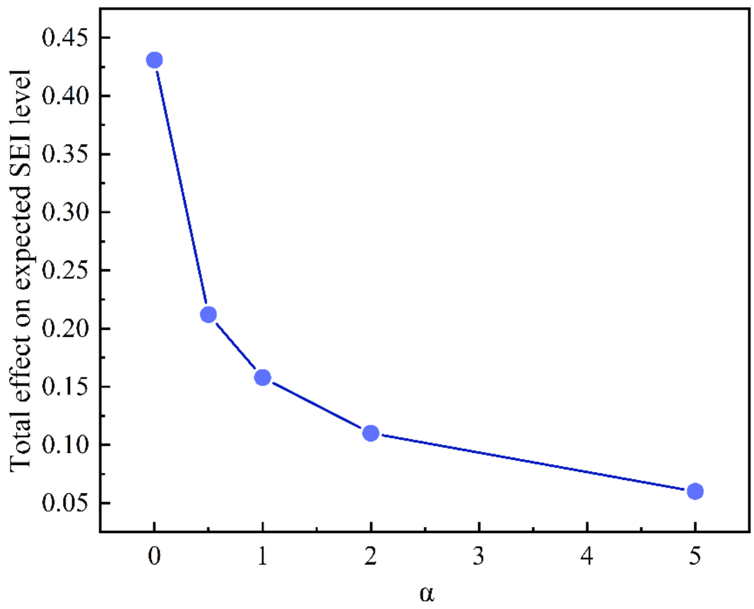

Figure S6. Sensitivity of Bayesian-network interventional effects to Laplace smoothing, Related to Figure 10.

Table S1. Summary of uncertainty sources and sensitivity or robustness checks across DTW, CCD, BN, and DBSCAN, related to STAR Methods.

Module	Uncertainty source	Baseline specification in this study	Sensitivity	Evidence reported
SEI weighting	Relative weight of Alignment vs Coordination	Equal weights (0.5, 0.5)	Weight perturbation from 0.3 to 0.7	Methods
SEI uncertainty	Sampling uncertainty across 113 units	Annual SEI summaries reported	Unit-level bootstrap, B = 1000, percentile 95% CIs	Table 2
DTW preprocessing	Influence of extreme DTW values on normalization	Alignment defined as 1 − normalized DTW	1–99% winsorization prior to normalization	Methods
BN structure	Structure learning variability	Hill-climbing with BIC	Bootstrap resampling for structural stability	Methods
BN inference	Prior regularization in discrete inference	Laplace smoothing used	Sensitivity to smoothing parameter α	Table S2 and Figure 11
DBSCAN zoning	Neighborhood parameterization and noise handling	Fixed setting ε = 0.1, MinPts = 8; noise retained as residual group	Inter-zone separation supported by ANOVA	Results
Local spatial tests	Multiple comparisons in LISA	Permutation tests with FDR control	BH-FDR at q = 0.10	Results

Note: DTW, Dynamic Time Warping; CCD, Coupling Coordination Degree; BN, Bayesian network; DBSCAN, Density-Based Spatial Clustering of Applications with Noise; CI, confidence interval; BH-FDR, Benjamini–Hochberg false discovery rate.

Table S2. Sensitivity of the carbon-axis total effect to Laplace smoothing, Related to Figure 11.

**α**	**TEC (E[S] diff)**
0	0.431
0.5	0.212
1	0.158
2	0.110
5	0.060

Note: α denotes the Laplace smoothing parameter. TEC, total effect on the carbon axis; E[S] diff, difference in the expected discretized SEI level.
